# Inhibition of IGF2BP1 attenuates renal injury and inflammation by alleviating m6A modifications and E2F1/MIF pathway

**DOI:** 10.7150/ijbs.78348

**Published:** 2023-01-01

**Authors:** Yan Mao, Feng Jiang, Xue-Jiao Xu, Lan-Bo Zhou, Rui Jin, Li-Li Zhuang, Chen-Xia Juan, Guo-Ping Zhou

**Affiliations:** 1 Department of Pediatrics, The First Affiliated Hospital, Nanjing Medical University, Nanjing, China.; 2 Department of Neonatology, Obstetrics and Gynecology Hospital of Fudan University, Shanghai, China.; 3 Department of Dermatology, Suzhou Hospital, Nanjing Medical University, Suzhou, China.; 4 Department of Nephrology, Jiangsu Province Hospital of Chinese Medicine, Affiliated Hospital of Nanjing University of Chinese Medicine, Nanjing, China.

**Keywords:** Transcriptional regulation, m6A RNA methylation, IGF2BP1, septic acute kidney injury, pyroptosis

## Abstract

Septic acute kidney injury (AKI) is characterized by inflammation. Pyroptosis often occurs during AKI and is associated with the development of septic AKI. This study found that induction of insulin-like growth factor 2 mRNA binding protein 1 (IGF2BP1) to a higher level can induce pyroptosis in renal tubular cells. Meanwhile, macrophage migration inhibitory factor (MIF), a subunit of NLRP3 inflammasomes, was essential for IGF2BP1-induced pyroptosis. A putative m6A recognition site was identified at the 3′-UTR region of E2F transcription factor 1 (E2F1) mRNA via bioinformatics analyses and validated using mutation and luciferase experiments. Further actinomycin D (Act D) chase experiments showed that IGF2BP1 stabilized E2F1 mRNA dependent on m6A. Electrophoretic mobility shift assay (EMSA) and chromatin immunoprecipitation (ChIP) indicated that E2F1 acted as a transcription factor to promote MIF expression. Thus, IGF2BP1 upregulated MIF through directly upregulating E2F1 expression via m6A modification. Experiments on mice with cecum ligation puncture (CLP) surgery verified the relationships between IGF2BP1, E2F1, and MIF and demonstrated the significance of IGF2BP1 in MIF-associated pyroptosis *in vivo*. In conclusion, IGF2BP1 was a potent pyroptosis inducer in septic AKI through targeting the MIF component of NLRP3 inflammasomes. Inhibiting IGF2BP1 could be an alternate pyroptosis-based treatment for septic AKI.

## Introduction

Acute kidney injury (AKI) caused by sepsis has a high death rate, which is associated with systemic inflammation 1. The leading causes of sepsis-induced AKI are pathogen-associated molecular patterns (PAMPs) and damage-associated molecular patterns (DAMPs) 2. Continuous stimulation would result in intracellular and extracellular environmental imbalance, immune cell infiltration, high oxidative stress, and cell death, ultimately leading to kidney injury with renal function and morphological alteration. Pyroptosis is a type of inflammatory cell death. In inflammatory disorders and malignancies, varieties of inflammasome formation, such as nod-like receptor protein 1 (NLRP1) inflammasomes, nod-like receptor protein 3 (NLRP3) inflammasomes, and absent in melanoma 2 (AIM-2) inflammasomes, could trigger cell pyroptosis. Pyroptosis activators like lipopolysaccharide (LPS), adenosine triphosphate (ATP), and oxidative stress can recruit NLRP3, apoptosis-associated speck-like protein containing a CARD (ASC), and cysteine-aspartic acid protease 1 (caspase1) 3. Then, caspase1 cleaves the protein gasdermin D (GSDMD) into Nand C-terminal fragments, creating holes in cell membrane, via which matured interleukin-1β (IL-1β) and interleukin-18 (IL-18) are released extracellularly 4. In renal diseases, NLRP3 pathway in pyroptosis has been extensively explored, providing a foundation for our investigation 5, 6.

RNA methylation on the sixth N atom of adenylate is defined as N6-methyladenosine (m6A), which is the most prevalent form of RNA internal modification 7. Recent research indicates that m6A level influences the stability of mRNA and effectiveness of mRNA translation and is therefore involved in various biological processes, including pyroptosis 8, 9. The m6A modification is reversible since enzymes such as methyltransferase-like 3 and 14 (METTL3 and METTL14) can catalyze it, as enzymes like fat mass and obesity-associated protein (FTO) can also erase it 10, 11. In contrast to these “writers” and “erasers”, “readers” are RNA-binding proteins that can recognize and direct m6A-modified RNAs for future processing 12, 13. Hence, these readers are the m6A executors. Insulin-like growth factor 2 mRNA-binding proteins (IGF2BPs), including IGF2BP1/2/3, were first identified in 1999 for their ability to bind to the 5'-UTR of insulin-like growth factor Ⅱ (IGF-Ⅱ) leader 3 mRNA 14. The first IGF2BP family member described is IGF2BP1 which is identified as a polysome-associated protein to stabilize c-MYC mRNA through binding to its coding region determinant (CRD) 15. It expresses in a broad range of fetal tissues and more than 16 cancers, but only in a limited number of normal adult tissues. IGF2BP1 is required for the transport of certain mRNAs and plays essential roles in embryogenesis, carcinogenesis, and chemo-resistance 16, 17 through affecting their stability, translatability, or localization 18, 19. Previous studies have pointed out that IGF2BP1, an m6A-reader, serves as an important oncogene in cancers by stabilizing or enhancing mRNA of its oncogenic factors 20, 21. Hence, IGF2BP1 may have great potential in cancer treatment 22. Apart from neoplastic diseases, IGF2BP1 also plays a crucial role in nonneoplastic diseases. For example, the elevation of IGF2BP1 was associated with macrophage infiltration into the atherosclerotic vascular wall 23. It has been reported to promote the NF-κB activation and the generation of pro-inflammatory cytokines in response to LPS 24, 25. Inhibiting IGF2BP1 could attenuate cardiomyocyte pyroptosis However, we only have limited evidence that IGF2BP1 could regulate pyroptosis in septic AKI. This study explored and confirmed the role of IGF2BP1 in septic AKI.

Here, we hypothesized that the elevation of IGF2BP1 in septic AKI caused renal damage and enhanced NLRP3 inflammasome activity via macrophage migration inhibitory factor (MIF) to exacerbate pyroptosis. The septic AKI model *in vivo* and *in vitro* was generated by performing cecum ligation puncture (CLP) surgery on mice or treating HK2 cells with LPS. In CLP mice, declined renal function was associated with the elevation in IGF2BP1 expression and pyroptosis. Further study demonstrated that E2F1 modified by IGF2BP1 transcriptionally promoted MIF-related pyroptosis though triggering the assembly of NLRP3 inflammasomes. Inhibiting IGF2BP1 might be an attractive and alternative pyroptosis-based treatment in the future for treating septic AKI.

## Methods and Materials

### Reagents and plasmids

In this study, lipopolysaccharide (LPS, #L2880) and MIF antagonist (ISO-1, #475837) were bought from Sigma-Aldrich (Missouri, USA). Cycloheximide (CHX, #HY-12320) and 3-Deazaadenosine (DAA, #HY-W013332) were purchased from MedChemExpress (MCE, New Jersey, USA). VX765 (#inh-vx765i) was purchased from Invivogen (California, USA). The sh-IGF2BP1, sh-METTL3, sh-MIF, sh-E2F1, and sh-NC were obtained from Genepharma company (Shanghai, China). Lentiviruses expressing sh-IGF2BP1 and nonsense control (NC) were purchased from Tsingke (Nanjing, China). The E2F1 overexpression vector was constructed using p-ENTER, while the overexpression plasmids for MIF or METTL3 were constructed in pcDNA3.1.

### AKI patients and controls

Patients with AKI admitted between December 2021 and July 2022 at The First Affiliated Hospital of Nanjing Medical University were included. The control participants were recruited from a healthy population that received a health checkup at The First Affiliated Hospital of Nanjing Medical University between February and July 2022. The critical elements of the clinic are listed in Table 1. The Institutional Review Board of The First Affiliated Hospital of Nanjing Medical University approved the study protocol (2022-SRFA-045). All patients provided informed permission prior to treatment.

### Mice model of AKI

The Committee on the Ethics of Animal Experiments at Nanjing Medical University approved the animal experiments (IACUC-2111039). Mice feeding and model building were performed in the animal biosafety level-3 laboratory (ABSL-3 Lab) of Nanjing Medical University. The male C57BL/6 J mice (weighting 25-30 g, 12-16 weeks of age) were purchased from Vital River Laboratory Animal Technology (Beijing, China) and randomly divided into groups. For the septic AKI model, mice were subjected to the CLP surgery 27 and euthanized 12 h later (n = 6 per group). In detail, after anesthetizing mice, a 1.2-1.8 cm incision was performed below the diaphragm to expose the cecum. After creating a ligation at the junction of the colon and cecum, a 22-gauge needle was used to puncture the cecum twice to squeeze out feces. Each mouse was fluid-resuscitated after the surgery. The Sham group accepted the incision without a cecum ligation puncture. ISO-1 could significantly inhibit the activity of MIF topoisomerase significantly 28, thus, each mouse was intraperitoneally administered according to body weight (20 μg/g of ISO-1 for the CLP+ISO-1 group and the same quantity of 1% DMSO for the CLP+DMSO group) 4-6 h prior to the CLP surgery. Injection of renal parenchyma (5×10^7^ TU/mouse) facilitated lentivirus-mediated gene transfer in the kidneys. The CLP operation was conducted one week after the injection. *In vivo* tests were conducted in accordance with Chinese regulations on the use and care of laboratory animals.

### Cell culture

Human Kidney-2 (HK2) cells were purchased from the Cell Bank of the Chinese Academy of Sciences (Shanghai, China) and cultured in DMEM/F12 (Gibco, USA) supplemented with 10% fetal bovine serum (FBS, Gibco, California, USA), 1% penicillin/streptomycin (Gibco, California, USA) at 37 °C with 5% CO_2_. LPS was dissolved in PBS and kept at -20 °C. HK2 cells were treated with serum-free DMEM/F12 for 8 h in advance and then replaced with new culture medium containing LPS of specified concentration or PBS solution of the same volume. To block protein synthesis, HK2 cells were incubated with 100 μM CHX 29 30 min prior to LPS/PBS incubation and then maintained for 12 h during LPS/PBS incubation. HK2 cells were pre-incubated with 20 ug/ml VX765 30 for 30 min to inhibit enzymes caspase-1 and its related pyroptosis. An m6A methylation inhibitor, DAA 31, was added to explore the effect of the total m6A level.

### Renal function and histology evaluation

The blood of mice was centrifuged at 1500 r/min at 4 °C, and the separated serum was stored at -80 °C. Serum creatinine (Scr) and BUN were detected to reflect renal function via commercialized kit reagents. Kidney tissues embedded with paraffin were cut into slices, followed by being immersed in 4% paraformaldehyde for hematoxylin and eosin (H&E) and periodic acid-Schiff (PAS) staining. The frozen kidney tissues were stored at -80 °C for protein or mRNA detection.

### Immunohistochemistry (IHC) and immunofluorescence staining

Before the immunostaining, antigen retrieval for paraffin-embedded tissues in a citrate antigen retrieval solution was conducted using microwave heating. For IHC, kidney tissues were incubated with antibodies against IGF2BP1 (#22803-1-AP, Proteintech, Wuhan, China), MIF (#20415-1-AP, Proteintech, Wuhan, China), or E2F1 (#ab288369, Abcam, Cambridge, UK). Immunofluorescence staining of kidney sections was performed with the antibody against IGF2BP1 (#22803-1-AP, Proteintech, Wuhan, China), while those of HK2 cells were performed with primary antibodies against MIF (#20415-1-AP, Proteintech, Wuhan, China), IGF2BP1 (#22803-1-AP, Proteintech, Wuhan, China), and NLRP3 (#AG-20B-0014-C100, AdipoGen, San Diego, USA). Cell nuclei were stained with DAPI. Immuno-stained samples were visualized using a Leica Thunder microscope system.

### Dot blot and Western blot (WB) analysis

Using the DynaBeads mRNA Purification Kit (ThermoFisher, Waltham, MA, USA), poly (A) RNA was purified from total RNA for dot blot. Poly(A) RNAs were diluted to concentrations of 250 ng/μl and 100 ng/μl. Each dilution was spotted twice (2 ul) on positively charged nylon membranes (Amersham Pharmacia, Piscataway, NJ, USA). The membranes with poly(A) RNAs were crosslinked using a Stratalinker 2400 Crosslinker. Then, the membranes were incubated for 5 min in wash buffer prior to 1-h blocking. The membranes were incubated in blocking buffer with diluted anti-m6A antibodies overnight at 4 °C. The levels of total m6A were detected using enhanced chemiluminescence after typical immunoblotting techniques including treatment with secondary antibodies.

WB was conventionally performed. The primary antibodies used were as follows: anti-IGF2BP1 (Proteintech, #22803-1-AP, Wuhan, China), anti-MIF (Proteintech, #20415-1-AP, Wuhan, China), anti-E2F1 (Abcam, #ab288369, Cambridge, UK), anti-NLRP3 (AdipoGen, #AG-20B-0014-C100, San Diego, USA), anti-Caspase1 (Cell signaling technology, #3866, Boston, Massachusetts, USA), anti-Cleaved-Caspase1 (Affinity, #AF4005, Jiangsu, China), anti-ASC (Affinity, #DF6304, Jiangsu, China), and anti-GAPDH (Abcam, #ab181602, Cambridge, UK).

### Methylated RNA immunoprecipitation-qPCR (MeRIP-qPCR) and co-immunoprecipitation (Co-IP)

For MeRIP-qPCR experiments, total RNA of HK2 cells with different treatments was extracted. After fragmentation, RNA was incubated with anti-m6A or IgG antibodies using the GenSeq® m6A MeRIP kit (Genseq, #GS-ET-001, Shanghai, China) for immunoprecipitation. Enrichment of m6A-containing mRNA was measured using a qPCR assay. For Co-IP experiments, collected HK2 cells were homogenized in lysis buffer from the IP/CoIP kit (#abs955, Absin, Shanghai, China) on ice. The cell lysates were co-incubated with selected antibodies at 4 °C overnight and then added with protein agarose A/G beads to reduce non-specific bindings. The precipitate was washed three times before WB. The antibodies employed were the same as those used in WB.

### Enzyme-linked immunosorbent assay (ELISA) and lactate dehydrogenase (LDH) release measurement

The thawing of samples was conducted at room temperature. Human IL-1β (#ab214025), IL-18 (#ab215539), Caspase 1 (#ab219633), IL-6 (#ab178013, Abcam, Cambridgeshire, UK), and TNF-α (#ab181421, Abcam, Cambridgeshire, UK) levels in the culture supernatant were measured with commercialized sandwich enzyme-linked kits from Abcam (Cambridgeshire, UK). Mouse MIF (#KE10027, Proteintech, Wuhan, China), Scr (#C011-2-1, Jiancheng, Nanjing, China), IL-1β (#KE10003, Proteintech, Wuhan, China) levels in mice were determined. Each sample was measured in quadruplicate or sextuplicate.

The extracellular LDH was examined to detect pyroptotic cell death using the LDH Cytotoxicity Assay Kit (#40209ES76, Yeasen, Shanghai, China). Briefly, the culture supernatant was centrifuged at 400 × g for 5 min and then loaded into a new 96‐well plate, mixed with 60 μL LDH detection working fluid. After incubation at room temperature for 30 min in the dark, the absorbance at 490 nm was detected using a microplate reader (Multiskan FC, Thermo Fisher Scientific, Waltham, MA, USA).

### Real-time quantitative PCR (qPCR) and cell viability analysis

For qPCR, total RNA was isolated using the EZNA ® Total RNA Kit I (R6834, Omega Bio-Tek, Norcross, GA, USA). cDNA was synthesized with the PrimeScript RT Master Mix kit (Takara, #RR036A, Kyoto, Japan). The relative level of mRNA expression was quantified with an SYBR Kit (Vazyme Biotech, #Q711, Nanjing, China) and normalized to that of GAPDH expression. Primers used for qPCR were listed in Table S1.

Cell viability was determined using the cell viability assay (MCE, #HY-K0301, New Jersey, USA). Briefly, HK2 cells were seeded into the 96-well plates and added with CCK-8 (10 ul/per well) for further incubation for 2 h at 37 °C. Optical density (OD) was detected at 450 nm using a microplate reader (Multiskan FC, Thermo Fisher Scientific, Waltham, MA, USA).

### Transient transfections and luciferase reporter assays

The MIF promoter was cloned into the pGL3-basic plasmids (Promega, Madison, WI, USA) for promoter study and named pGL3-MIF. The wild-type (Wt) and mutant (Mut) PCR products containing the E2F1 binding motif in the MIF promoter were synthesized and cloned into the pGL3-basic plasmids (Generay Biotech Ltd, Shanghai, China), which were named E2F1-Wt and E2F1-Mut respectively. To explore the mechanism by which IGF2BP1 stabilized E2F1 mRNA via the m6A site in the 3'-UTR region, adenosine (A) in the m6A motif was replaced by cytosine (C) and then cloned into pmir-GLO reporter plasmids using Tsingke (Nanjing, China). Transfections were carried out using Lipofectamine-3000 transfection reagent (Invitrogen, Carlsbad, CA, USA). Each luciferase-containing plasmid (400 ng) and internal control plasmid (pRL-TK, Promega, Madison, WI, USA; 4 ng) were co-transfected. The dual-luciferase reporter gene assay kit (Promega, Madison, WI, USA) was applied to detect luciferase activities. The activities of firefly luciferase were normalized to those of Renilla luciferase.

### Electrophoretic mobility shift assay (EMSA)

For EMSA, the Wt and Mut short oligonucleotide probes were synthesized by GenePharma (Shanghai, China). The probes were biotin end-labeled using the Biotin 3' End DNA Labeling Kit (Thermo Pierce, Massachusetts, USA). The labeled and unlabeled probes were annealed to double-stranded probe DNA. EMSA assays were carried out using the LightShift Chemiluminescent EMSA kit (Thermo Fisher Scientific, Massachusetts, USA). For the super shift assay, anti-E2F1 (Abcam, #ab288369, Cambridge, UK) antibody or IgG (Cell signaling technology, #3900, Boston, Massachusetts, USA) were added to the nuclear extracts. All mixtures were separated on 6% native polyacrylamide and then transferred onto positively charged nylon membranes (Amersham Pharmacia, Piscataway, NJ, USA). After crosslinking the DNA by UV, the biotin-labeled DNA was identified using chemiluminescence. Probe sequences used for EMSA were listed in Table S1.

### Chromatin immunoprecipitation (ChIP)

ChIP was conducted using a Magna ChIP® A/G Chromatin Immunoprecipitation Kit (Merck, #17-10086, Darmstadt, Germany). Protein-DNA complexes were incubated with anti-E2F1 (Proteintech, #66515-1-Ig, Wuhan, China) or IgG (Merck, #17-10086, Darmstadt, Germany) antibodies coupled with protein beads at 4 ℃ overnight. Next, DNA was purified and precipitated for subsequent PCR analysis after elution and reverse cross-linking. The PCR was performed using Rapid Taq Master Mix (Vazyme, #P222-01, Nanjing, China). On a 2% agarose gel, the products were separated by electrophoresis. 10% of the initial sheared chromatin DNA served as the input control after reverse cross-linking and purification. In addition, ChIP DNA was detected by quantitative PCR using an SYBR Kit (Vazyme Biotech, #Q711, Nanjing, China) using the StepOnePlus™ Real-Time PCR System (Applied Biosystems, Foster City, CA, USA). Primers used for ChIP were listed in Table S1.

### Bioinformatics

Database searches to find homologies between sequences were performed using the BLAST algorithm (http://blast.ncbi.nlm.nih.gov/Blast.cgi) 32. The FPROM, TSSG, and TSSW algorithms (http://www.softberry.com/berry.phtml?topic=index&group=programs&subgroup=promoter) 33 and Promoter 2.0 (https://services.healthtech.dtu.dk/service.php?Promoter-2.0) 34 website were used to predict a promoter sequence from the 5' end of the MIF gene. The E2F1 binding site within the MIF promoter was predicted by JASPAR (https://jaspar.genereg.net/) 35 and UCSC (http://genome.ucsc.edu/) 36 databases. Analysis of ChIP-seq data was performed using the Cistrome Data Browser website (http://cistrome.org/db) 37. We investigated the m6A peak in E2F1 mRNA using a database named RMVar (https://rmvar.renlab.org/) 38.

### Statistical analysis

GraphPad Prism software (version 7.00) was used for statistical analyses and data visualization, with each point representing independent data. *P* values were calculated with t-test, one-way ANOVA, two-way ANOVA, and Pearson analysis. Data were presented as means ± SEMs from three independent experiments. The *P* values (*P < 0.05, **P < 0.01 and ***P < 0.001) were considered statistically significant while N.S. meant non-significance.

## Results

### IGF2BP1 is a pyroptosis inducer in septic AKI

**Figure 1:**
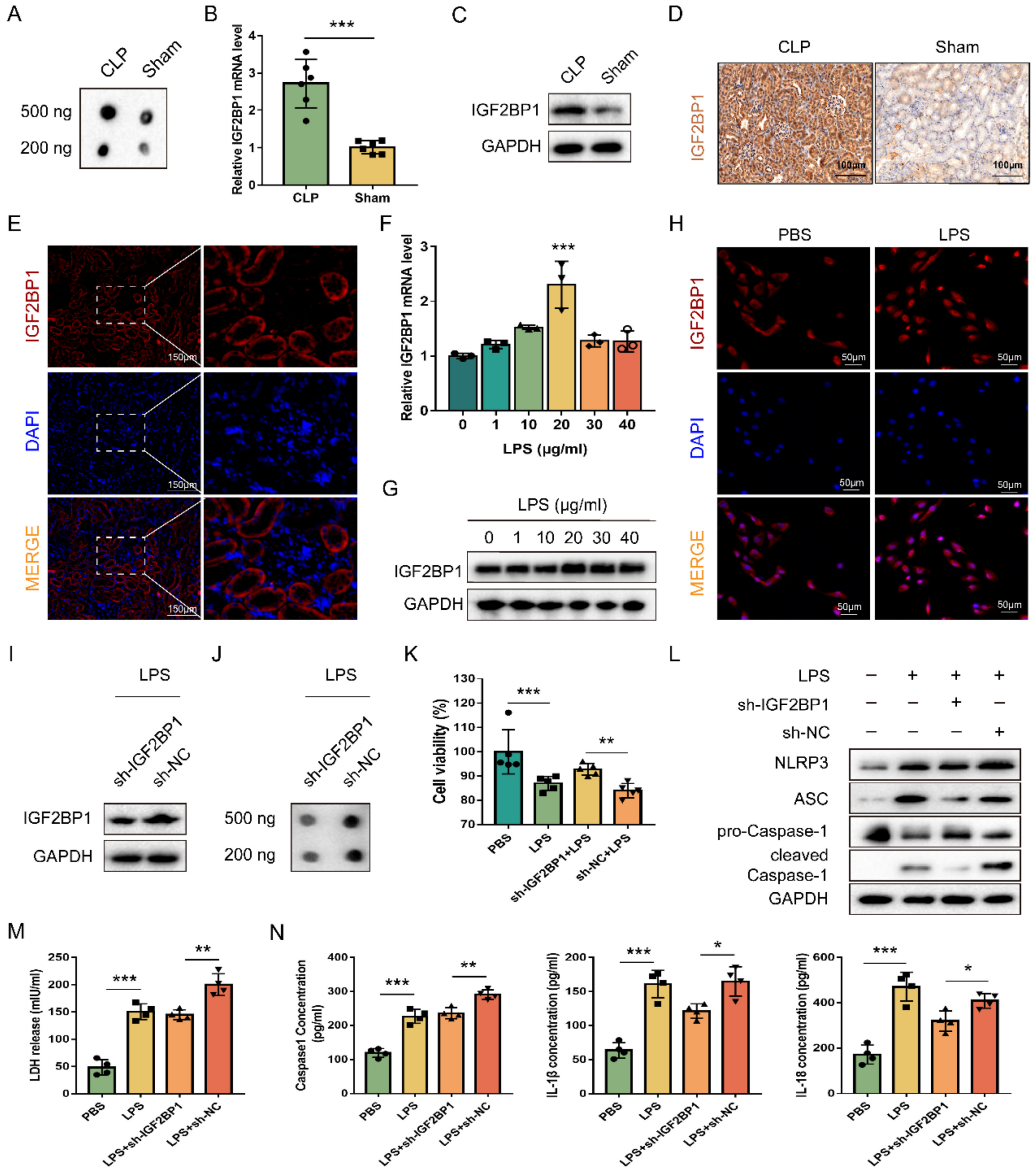
** IGF2BP1 is a pyroptosis inducer in septic AKI.** (**A**) Total m6A levels in CLP and Sham mice, as measured by dot blot. (**B-D**) IGF2BP1 mRNA and protein levels in CLP and Sham mice, as measured by qPCR (B), WB (C), and IHC (D; Scale bar, 100 μm). (**E**) Location of IGF2BP1 protein in the kidney tissue from mice, as measured by immunofluorescence. Immunofluorescent staining of IGF2BP1 (red) and nuclear staining with DAPI (blue) are shown. Scale bar, 150 μm. (**F-H**) Induction of IGF2BP1 by the indicated concentration of LPS for 12 h in HK2 cells, as measured by qPCR (F), WB (G), and immunofluorescence (H; Scale bar, 50 μm). Immunofluorescent staining of IGF2BP1 (red) and nuclear staining with DAPI (blue) are shown. (**I**) IGF2BP1 protein expression in sh-IGF2BP1/sh-NC-expressing HK2 cells following treatment with the indicated concentration of LPS for 12 h, as measured by WB. (**J**) Total m6A level in sh-IGF2BP1/sh-NC-expressing HK2 cells following induction with or without LPS for 12 h, as measured by dot blot. (**K-N**) Pyroptosis levels in sh-IGF2BP1/sh-NC-expressing HK2 cells following induction with or without LPS for 12 h. Cell viability (K), pyroptosis-related proteins (L), LDH release (M), secretory Caspase-1, IL-1β, and IL-18 levels (N) were measured. Statistical analysis was performed using a t-test (B, K, M, N) and one-way ANOVA (F). *P < 0.05, **P < 0.01 and ***P < 0.001.

Worse renal function in the CLP mice was confirmed as the serum creatinine (Scr) level was much higher in the CLP mice than in the Sham ones (Figure S1A). Total m6A levels were elevated more in the kidney tissues of CLP mice than in those from Sham groups (Figure 1A). To explore the expression levels of m6A regulators in AKI, we determined their mRNA levels via qPCR (Figure S1B) and found that IGF2BP1 was high-expressed in CLP mice (Figure 1B). Next, IGF2BP1 level was measured through WB, which was 2.68-fold higher in the CLP group than in the Sham group (Figure 1C). The IHC showed the same trend in the IGF2BP1 expression (Figure 1D). The results indicated that IGF2BP1 played a certain role in sepsis AKI. As shown in Figure 1E, it was evident that IGF2BP1 mainly located in renal tubular cells rather than in glomerular cells. Hence, HK2 cells were chosen for further investigation. The intracellular IGF2BP1 levels were evaluated to determine the optimal LPS dose. Based on preliminary experiments, HK2 cells were treated for 12 h with varying doses of LPS (0, 1, 10, 20, 30, and 40 μg/ml). As shown in Figure 1F-1H, the peak expression level of IGF2BP1 appeared at 20 μg/ml LPS stimulation at both mRNA and protein levels. At the same time, the mRNA of inflammatory factors, mainly IL-6 and TNF-α, showed peak levels (Figure S2A). The secretory IL-6 and TNF-α in the supernatants of HK2 cells treated with the indicated concentration of LPS for 12 h increased significantly (Figure S2B), confirming the inflammatory state of LPS-stimulated HK2 cells. Thus, the stimulation of 20 μg/ml LPS for 12 h was considered as the optimal concentration for studying the involvement of IGF2BP1 *in vitro*.

To explore the relationship between IGF2BP1 and pyroptosis in LPS-stimulated HK2 cells, the IGF2BP1 gene was knocked down by shRNA, and verified via WB (Figure 1I). The total m6A level was down-regulated after the knockdown of IGF2BP1 (Figure 1J), suggesting that the m6A modification levels induced by LPS in HK2 cells were realized via IGF2BP1. Notably, when compared with those stimulated by PBS, the induction of IGF2BP1 in HK2 cells by LPS promoted pyroptosis, as shown by cell viability, pyroptosis-related protein levels, LDH release, and secretory Caspase-1, IL-1β, and IL-18 levels (Figure 1K-N). The knock-down of IGF2BP1 also improved the cell viability of HK2 stimulated by LPS (Figure 1K). NLRP3, ASC, and Caspase1 p20 were all down-regulated in the sh-IGF2BP1-expressing HK2 cells (Figure 1L). LDH release levels, secretory Caspase-1, IL-1β, and IL-18 levels were reduced as the IGF2BP1 expression level was down-regulated (Figure 1M & 1N). The results suggested that the knockdown of IGF2BP1 reduced pyroptosis in LPS-stimulated HK-2 cells, further confirming that IGF2BP1 was a pyroptosis inducer in septic AKI.

### MIF is required for IGF2BP1-induced pyroptosis in AKI

**Figure 2:**
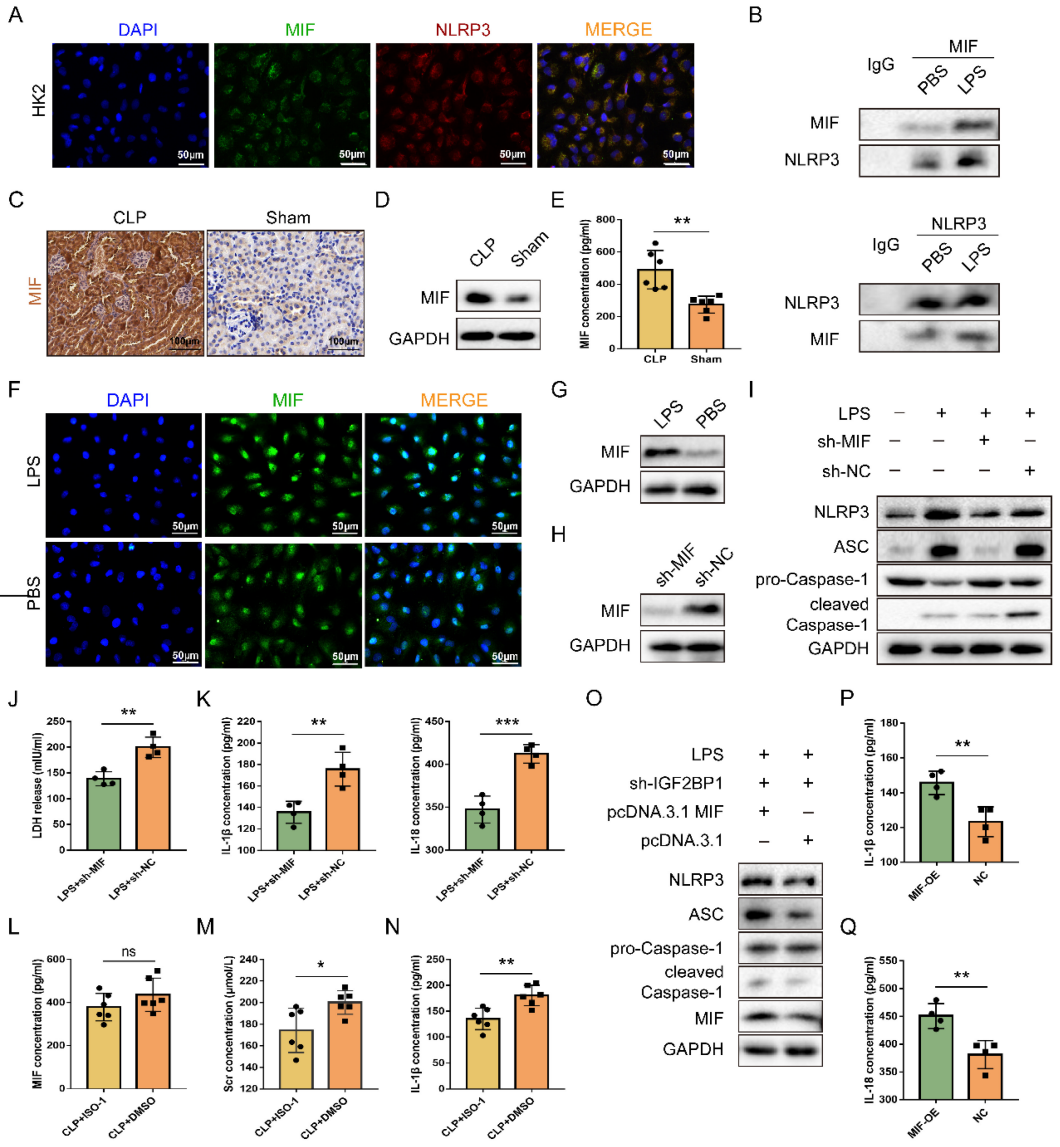
** IGF2BP1 induces pyroptosis via targeting MIF.** (**A & B**) MIF interacted with NLRP3. The co-localization of MIF protein and NLRP3 protein in HK2 cells was measured by immunofluorescence (A). Immunofluorescent staining of NLRP3 (red), MIF (green), and nuclear staining with DAPI (blue) are shown. Scale bar, 50 μm. The combination of MIF protein and NLRP3 protein in HK2 cells with or without LPS stimulation was measured by co-IP (B). (**C-E**) MIF protein levels in the CLP and Sham mice, as measured by IHC (C; Scale bar, 100 μm), WB(D), and ELISA (E). (**F & G**) MIF protein levels in HK2 cells treated with the indicated concentration of LPS for 12 h, as measured by immunofluorescence (F; Scale bar, 50 μm) and WB (G). Immunofluorescent staining of MIF (green) and nuclear staining with DAPI (blue) are shown. (**H**) MIF protein levels in sh-MIF/sh-NC-expressing HK2 cells treated with the indicated concentration of LPS for 12 h, as measured by WB. (**I-K**) Pyroptosis levels in sh-MIF/sh-NC-expressing HK2 cells following the induction with or without the indicated concentration of LPS for 12 h. Pyroptosis-related proteins (I), LDH release (J), secretory IL-1β, and IL-18 levels (K) were measured. (**L-N**) Serum MIF (L), creatinine (M), and IL-1β (N) levels in CLP mice treated with or without ISO-1, as measured by ELISA. (**O-Q**) Pyroptosis level in sh-IGF2BP1-expressing HK2 cells with MIF over-expression following the induction with the indicated concentration of LPS for 12 h. Pyroptosis-related proteins (O), secretory IL-1β level (P), and secretory IL-18 level (Q) were measured. Statistical analysis was performed using a t-test (E, K-N, P-Q). *P < 0.05, **P < 0.01 and ns, non-significance.

Pyroptosis can be reduced by inhibiting MIF, which could activate NLRP3 inflammasomes 39. We thereby hypothesized that pyroptosis induced by IGF2BP1 in AKI was realized via targeting MIF. As shown in Figure 2A, MIF co-localized with NLRP3 in HK2 cells. Meanwhile, the combination of MIF protein and NLRP3 protein existed not only in control HK2 cells but also in LPS-stimulated HK2 cells (Figure 2B). It further confirmed the interaction between MIF and NLRP3 in septic AKI. We then examined whether IGF2BP1 affects the expression of MIF in HK2 cells. As expected, MIF was up-regulated in CLP mice (Figure 2C-2E) and LPS-induced HK2 cells (Figure 2F & 2G).

Despite the fact that LPS up-regulated MIF, whether the MIF was required for IGF2BP1-induced pyroptosis in septic AKI remained unknown. To address this, MIF was knocked down in HK2 cells following induction of IGF2BP1 (Figure 2H). It was evident that knocking down MIF could partly abolish IGF2BP1-induced pyroptosis (Figure 2I-2K). ISO-1, an inhibitor of MIF 40, 41, was found to alleviate kidney injury 42, 43 and reduce the pyroptosis caused by MIF in AKI 44. For confirming the role of the MIF in pyroptosis in the septic AKI, mice were injected with ISO-1 to inhibit MIF activity (Figure 2L), with the CLP + DMSO group to serve as a control group. As shown in Figure 2M, Scr in CLP + ISO-1 group was decreased in contrast with that in CLP + DMSO group (174.3 ± 8.409 μmol/L vs. 200.2 ± 4.383 μmol/L). Meanwhile, by inhibiting MIF with ISO-1, pyroptosis-associated IL-1β release in CLP mice was also inhibited (Figure 2N), suggesting that IGF2BP1-induced pyroptosis in septic AKI was realized via MIF. Furthermore, at basal level, overexpression of MIF in sh-IGF2BP1 HK2 cells aggravated pyroptosis (Figure 2O-2Q). Hence, MIF was a target of IGF2BP1 required for LPS-induced pyroptosis.

### MIF is transcriptionally promoted by IGF2BP1

**Figure 3:**
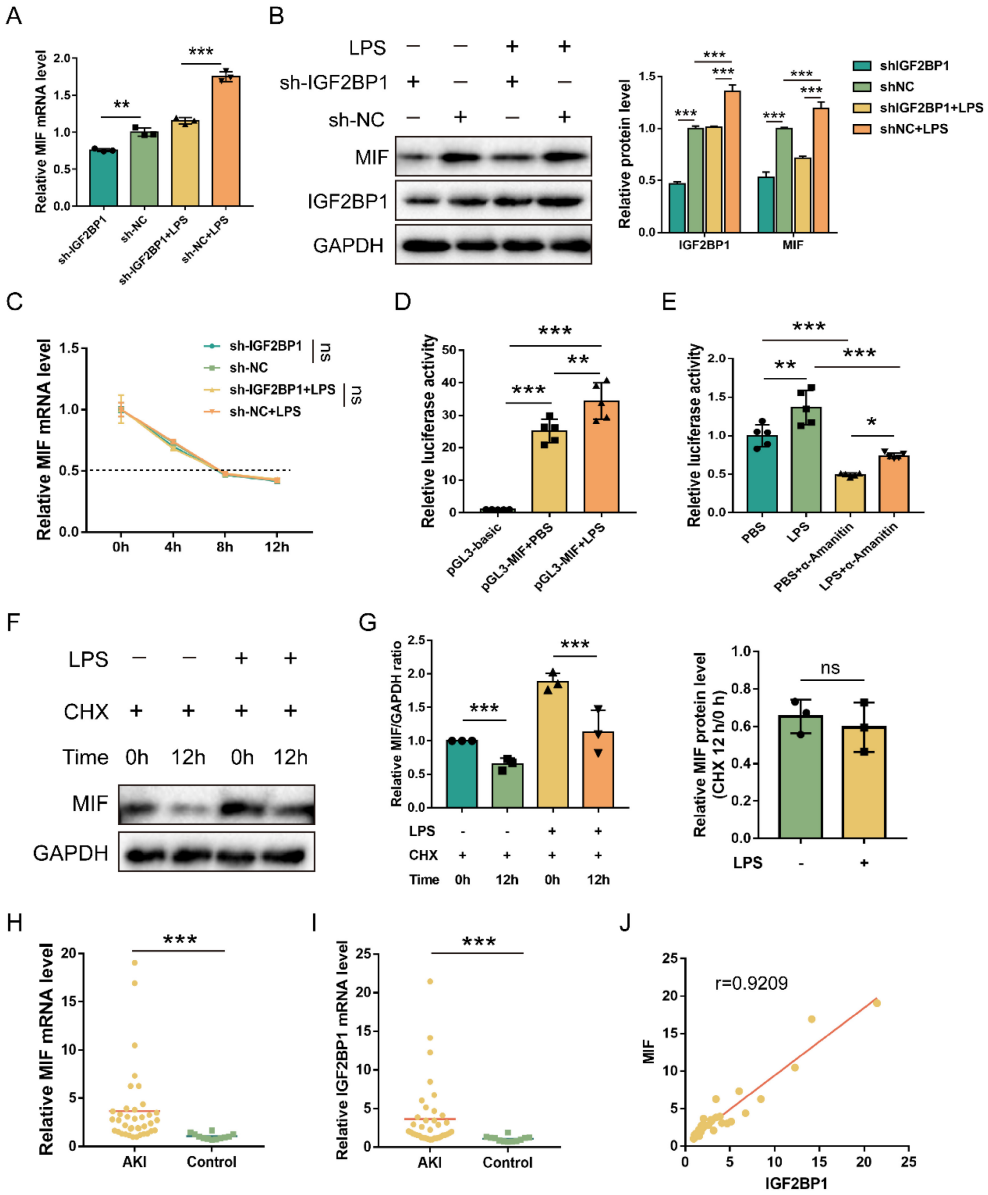
** MIF is transcriptionally promoted by IGF2BP1.** (**A**) MIF mRNA levels in sh-IGF2BP1/sh-NC-expressing HK2 cells following treatment with or without LPS for 12 h, as measured by qPCR (A). (**B**) MIF protein levels in sh-IGF2BP1/sh-NC-expressing HK2 cells following treatment with or without LPS for 12 h, as measured by WB. The quantification of the relative MIF protein levels was shown in the right panel. (**C**) Actinomycin D (Act D) chase experiments for MIF mRNA expression in sh-IGF2BP1/sh-NC-expressing HK2 cells following treatment with or without the indicated concentration of LPS for 12 h. (**D**) MIF promoter activities in HK2 cells following the treatment with or without LPS for 12 h. (**E**) MIF promoter activities in HK2 cells in the absence or presence of α-Amanitin, as measured by luciferase assays. (**F & G**) IGF2BP1 did not affect the protein stability of MIF. HK2 cells were treated with CHX (100 μM) during administration with or without LPS for 12 h. The ratio between MIF and GAPDH was graphed in the middle panel, and the relative MIF level with or without CHX was also calculated in HK2 cells treated with or without LPS (right panel). (**H**) MIF mRNA levels in AKI patients (n=38) and control samples (n=12), as measured in qPCR. (**I**) IGF2BP1 mRNA levels in AKI patients (n=38) and control samples (n=12), as measured in qPCR. (**J**) Correlation between IGF2BP1 mRNA and MIF mRNA in AKI patients (n=38) and control samples (n=12), as analyzed by Pearson analysis. Statistical analysis was performed using one-way ANOVA (A, D, E), two-way ANOVA (B, C), t-test (G), Wilcoxon rank sum test (H, I), and Pearson analysis (J). **P < 0.01, ***P < 0.001, and ns, non-significance.

Previous results showed that knocking down IGF2BP1 downregulated total m6A level in LPS-induced HK2 cells (Figure 1J). MIF expression was then determined in the sh-IGF2BP1/sh-NC-expressing HK2 cells, and we found that the knockdown of IGF2BP1 down-regulated MIF mRNA level in septic AKI (Figure 3A). Meanwhile, as shown in Figure 3B, knocking down IGF2BP1 decreased in MIF protein level. The elevation of MIF caused by LPS was reduced by the IGF2BP1 knockdown. Hence, IGF2BP1 was essential for promoting MIF expression in septic AKI. These results suggested that IGF2BP1 promoted MIF via its m6A-reading function. We further explored whether the mRNA and protein levels of MIF were concurrently elevated by IGF2BP1 in septic AKI, and examined the half-life of the remaining MIF mRNA in cells treated with actinomycin D (Act D), which is a pan-RNA synthesis inhibitor 45. We found that the induction of IGF2BP1 did not affect the stability of MIF mRNA (Figure 3C). However, the activity of MIF promoter (-2000/+200 relative to TSS) was positively regulated by IGF2BP1, which was about 1.37-fold of that in the PBS group (Figure 3D). The mechanism underlying IGF2BP1 promoting the transcription of MIF was confirmed by the inhibition of the elevated MIF promoter activity through inhibiting RNA polymerase II with α-amanitin (Figure 3E). We also assessed the possibility whether IGF2BP1 could enhance MIF at the protein level, but found IGF2BP1 did not affect protein stability of MIF (Figure 3F & 3G). IGF2BP1 only regulated the transcription of MIF. The upregulation of the MIF protein may have been resulted from the prior promotion of its mRNA.

Subsequently, we analyzed the correlation between MIF and IGF2BP1 in AKI samples. MIF and IGF2BP1 were upregulated in the AKI patients compared to the control ones (Figure 3H & 3I). Further analysis demonstrated that MIF mRNA levels were positively correlated with the IGF2BP1 mRNA levels (Figure 3J), indicating that IGF2BP1 might promote MIF* in vivo*.

### E2F1 is required for IGF2BP1 to stimulate the transcription of MIF

**Figure 4:**
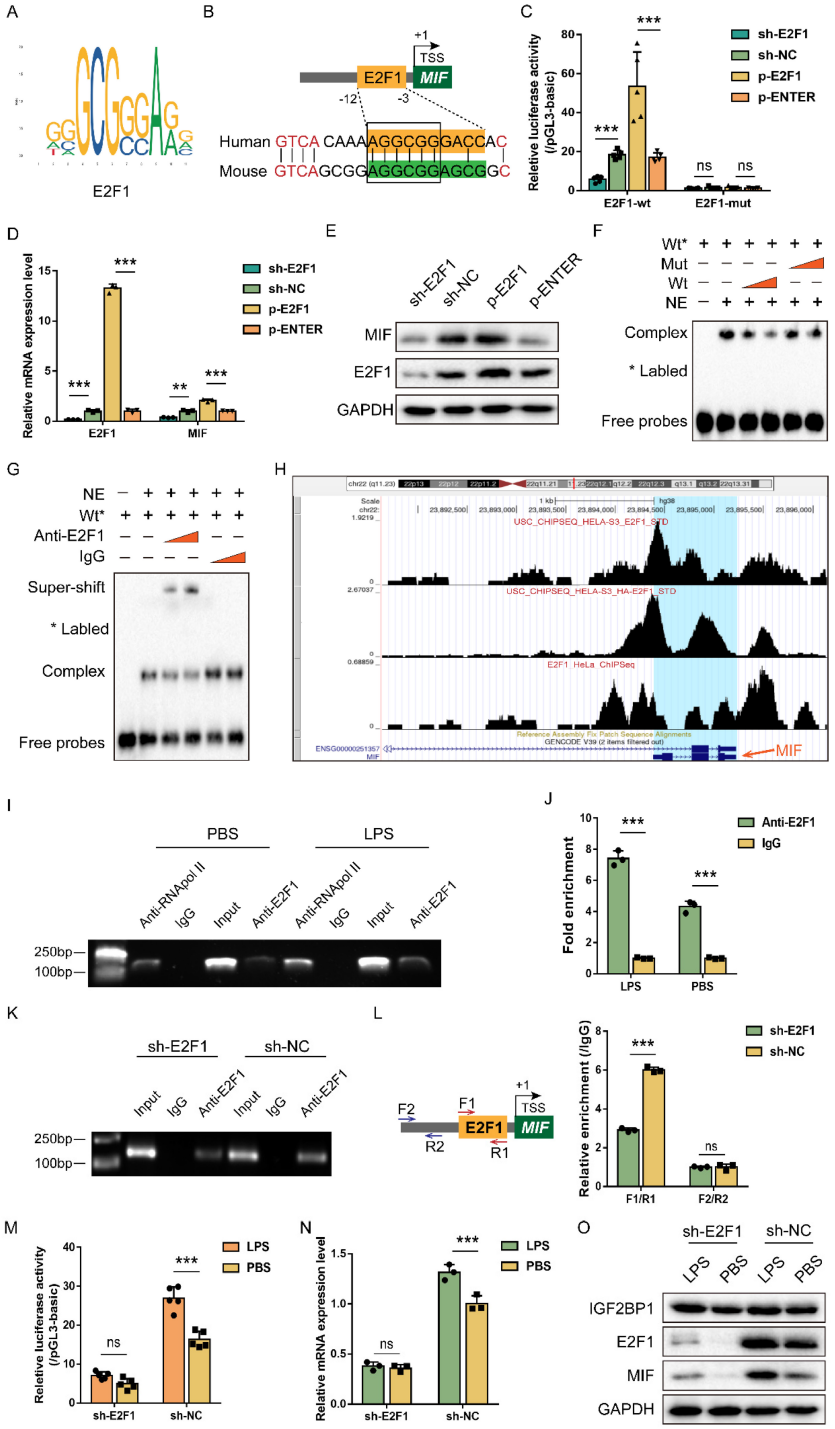
** IGF2BP1 promotes MIF transcription via E2F1.** (**A**) The specific binding site bases of transcription factor E2F1, downloaded from the JASPAR website (https://jaspar.genereg.net/). (**B**) The conserved E2F1 binding motif of the MIF promoter between humans and mice. (**C**) MIF promoter activities with or without intact E2F1 motif with E2F1 overexpressed or knocked down in HK2 cells, as measured by luciferase assay. (**D & E**) MIF mRNA and protein levels with E2F1 overexpressed or knocked down in HK2 cells, as measured by qPCR (D) and WB (E) in HK2 cells respectively. (**F**) The interaction between probes compassing the E2F1 motif and nuclear exacts (NE) from HK2 cells, as measured by EMSA. The proportion of wild-type (Wt) probes and mutant (mut) probes in each assay was different. (**G**) The interaction between probes compassing the E2F1 motif and nuclear exacts (NE) from HK2 cells, as measured by EMSA. The type or dose of antibodies was different for each assay. (**H**) ChIP-seq of E2F1 related to MIF in HeLa cells, as shown on the Cistrome Data Browser website (http://cistrome.org/db). (**I & J**) ChIP assays using anti-E2F1 antibody and control IgG antibody in HK2 cells with or without the indicated concentration of LPS for 12 h (I). QPCR was used to quantify immunoprecipitated chromatin fragments using primers specific to E2F1 binding sites (J). (**K & L**) ChIP assays using anti-E2F1 antibody and IgG antibody in HK2 cells with E2F1 knocked down (K). qPCR was used to quantify immunoprecipitated chromatin fragments (L). F1/R1 region compassed the E2F1 motif in the MIF promoter, while F2/R2 region represented an unrelated region in the MIF promoter. (**M-O**) IGF2BP1 promotes MIF promoter activities and expression via E2F1 in HK2 cells. MIF promoter activities, mRNA levels, and protein levels in sh-E2F1/sh-NC-expressing HK cells with or without inducible IGF2BP1 expression, as measured by luciferase assay (M), qPCR (N), and WB (O) respectively. Statistical analysis was performed using one-way ANOVA (C, D) and t-test (J, L-N). **P < 0.01, ***P < 0.001, and ns, non-significance.

Since IGF2BP1 is not a transcription factor and cannot directly alter MIF transcription, we explored the transcription factor responsible for the action of IGF2BP1. To confirm the central promoter position, we used FPROM, PROMOTER 2.0, TSSG, and TSSW online software and finally chose a total of 800 bp (-600/200 relative to TSS) for further analysis (Figure S3). JASPAR and UCSC online tools were introduced to predict probable transcription factor binding in the region, which revealed an E2F1 motif that is conserved across humans and mice (Figure 4A & 4B). Validation of the binding motif in Norway rat, zebrafish, and pig showed that it did not remain conserved in these species (Figure S4). Then, we mutated the E2F1 motif to analyze whether E2F1 was required for the activity of the MIF promoter. As expected, overexpressing E2F1 promoted MIF promoter activity, while knocking E2F1 down reduced MIF promoter activity (Figure 4C). This impact was inhibited, when the E2F1 motif was altered (Figure 4C), indicating that E2F1 was necessary for the MIF promoter activity. Changes in MIF mRNA and protein levels were consistent with findings from its promoter (Figure 4D&4E). EMSA revealed that the binding of E2F1 protein to probes containing E2F1 motif in the MIF promoter was reduced in a dose-dependent manner by unlabeled probes but not by mutant probes without the intact motif (Figure 4F). Co-incubation with anti-E2F1 antibodies during super-shift assays further validated the binding between E2F1 and the MIF promoter (Figure 4G). E2F1 has been identified as a transcription factor that binds to the promoter of MIF in HeLa cells 46, 47, as shown on the Cistrome Data Browser website (Figure 4H). ChIP was conducted using HK2 cells with or without LPS for 12 h, and we found that the binding of E2F1 and the relative motif in the MIF promoter was enhanced by LPS (Figure 4I&4J). When E2F1 was knocked down, the binding of E2F1 to the F1/R1 region that encompassed the motif was diminished (Figure 4K). Whether E2F1 was inhibited or not, adjacent F2/R2 region exhibited no E2F1 binding (Figure 4L). We further examined whether IGF2BP1 stimulated MIF transcription and translation through E2F1. In HK2 cells with knockdown of E2F1, induction of IGF2BP1 failed to increase MIF promoter activity, mRNA and protein production of MIF (Figure 4M-4O), further demonstrating that E2F1 was required for IGF2BP1 to promote the expression of MIF.

### IGF2BP1 stabilizes E2F1 mRNA and promotes pyroptosis in an m6A-dependent manner

**Figure 5:**
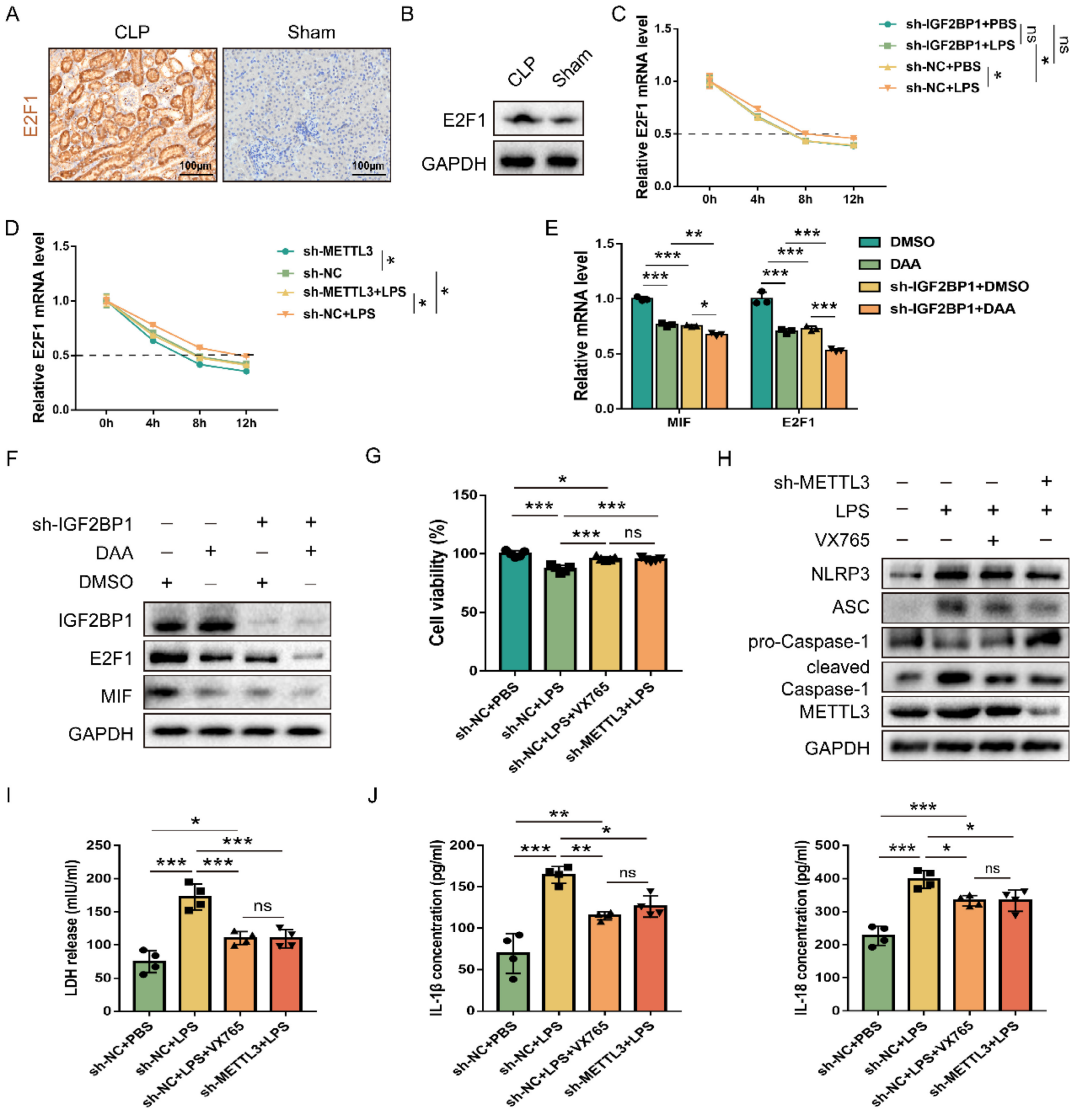
** IGF2BP1 m6A-dependently stabilizes E2F1 mRNA.** (**A & B**) E2F1 protein expression in the CLP and Sham mice, as measured by IHC (A; Scale bar, 100 μm) and WB (B). (**C**) Act D chase experiments for E2F1 mRNA in HK2 cells with or without knockdown of IGF2BP1 following treatment with or without LPS for 12 h. (**D**) Act D chase experiments for E2F1 mRNA in HK2 cells with or without knockdown of METTL3 following treatment with or without LPS for 12 h. (**E & F**) The mRNA and protein levels of E2F1 and MIF were measured using qPCR (F) and WB (G) in HK2 cells with IGF2BP1 knocked down, treated with or without DAA for 12 h. (**G**) Cell viabilities of sh-METTL3/sh-NC-expressing HK2 cells with the treatment of LPS in the absence or presence of VX765, as measured by CCK-8 assay. (**H-J**) Pyroptosis level in sh-METTL3/sh-NC-expressing HK2 cells with or without the indicated concentration of LPS in the absence or presence of VX765. Pyroptosis-related proteins (H), LDH release (I), secretory IL-1β and IL-18 levels (J) were measured. Statistical analysis was performed using two-way ANOVA (C-E) and one-way ANOVA (G, I, J). *P < 0.05, **P < 0.01, ***P < 0.001, and ns, non-significance.

E2F1 increases MIF transcription and is necessary for IGF2BP1 to promote MIF (Figure 4); hence, we studied whether IGF2BP1 promoted E2F1. E2F1 levels were increased in mice with septic AKI (Figure 5A & 5B). As it was reported that IGF2BP1 regulated mRNA stability through m6A modification in cells 48 and IGF2BP1 could serve as a post-transcriptional enhancer of the E2F-driven hallmark 49, we then examined whether IGF2BP1 also stabilized E2F1 mRNA in septic AKI. Act D chase assays revealed that IGF2BP1 stabilized E2F1 mRNA in HK2 cells with induced IGF2BP1 (Figure 5C).

Given that IGF2BP1 is widely considered as a reader for m6A modifications, we investigated whether m6A mediated IGF2BP1 involvement in elevating E2F1 mRNA stability in septic AKI. After knocking down METTL3, a major m6A writer, we found that control cells exhibited greater E2F1 mRNA stability than sh-METTL3-expressing HK cells (Figure 5D), indicating that METTL3 expression was positively correlated with E2F1 mRNA stability. In addition, the induction of IGF2BP1-increased E2F1 mRNA stability was reduced after knocking down METTL3 (Figure 5D). As demonstrated by the fact that METTL3 regulated total m6A RNA methylation 50, 51 and combined with the results shown in Figure 5D, we found that IGF2BP1 might stabilize E2F1 mRNA in an m6A-dependent manner in septic AKI.

In order to further confirm that IGF2BP1 affected MIF in an m6A-dependent manner, we introduced DAA, an S-adenosylhomocysteine hydrolase inhibitor and an m6A methylation inhibitor 52, 53. When HK2 cells were treated with DAA, the mRNA and protein levels of MIF and E2F1 were significantly lower than their baseline values (Figure 5E & 5F). Moreover, the decrease of MIF and E2F1 expression resulted from the knockdown of IGF2BP1 were enhanced in the presence of DAA (Figure 5E & 5F). Further analyses showed that the decline in E2F1 and MIF expression by DAA was reversed when the IGF2BP1 was overexpressed (Figure S5). We then tested whether METTL3-mediated m6A RNA methylation is likewise required for IGF2BP1 to generate pyroptosis since IGF2BP1 induced pyroptosis via promoting MIF transcription. By potently inhibiting the caspase-1 enzyme, VX765 might prevent decreased cell viability 54, 55. METTL3 inhibition had the same effect as simultaneous dosing with VX765 (Figure 5G). We discovered that knocking down METTL3 reduced pyroptosis in HK2 cells (Figures 5H-5J), suggesting that IGF2BP1-associated pyroptosis is likewise m6A-dependent. Since blocking E2F1-mediated transcription of MIF is necessary for IGF2BP1 to trigger pyroptosis (Figure 2-4), the m6A-dependent stability of E2F1 mRNA may be the rate-limiting step in the whole process. Thus, we concluded that the regulation of E2F1, MIF, and related pyroptosis by IGF2BP1 is in an m6A-dependent manner.

### E2F1 mRNA is m6A methylated and stabilized by IGF2BP1

**Figure 6:**
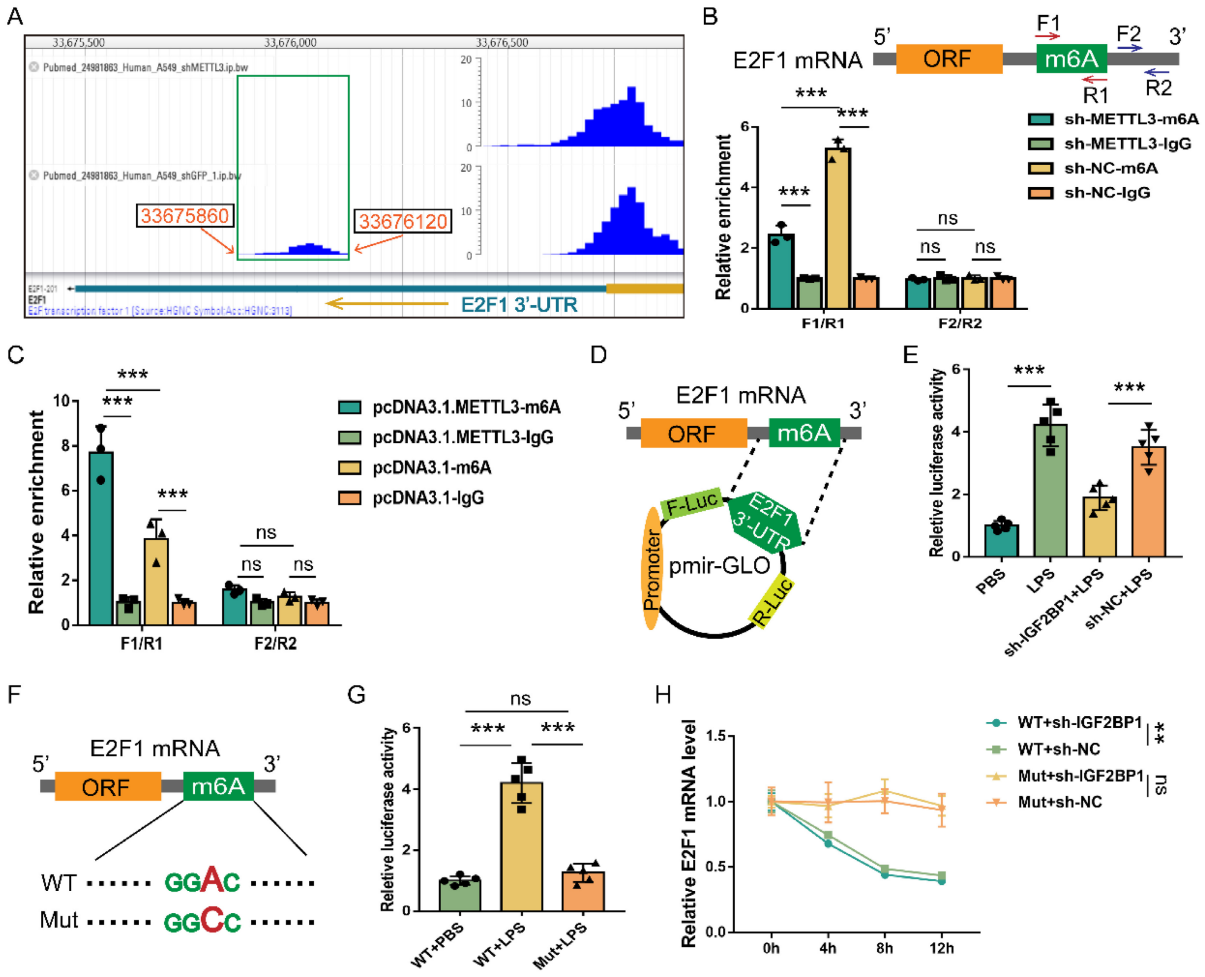
** E2F1 mRNA is m6A methylated and stabilized by IGF2BP1.** (**A**) DART-seq data from the GSE54365 set analyzed on the RMVar website (https://rmvar.renlab.org/), which was based on the A549 cells with or without knockdown of METTL3 and revealed an m6A peak within the 3′-UTR region of E2F1 mRNA. (**B & C**) The enrichment of E2F1 mRNA in RIP experiments in HK2 cells with or without METTL3 knockdown (B) and with or without METTL3 overexpression (C). F1/R1 region encompassed the potential m6A site in the 3′-UTR region of E2F1 mRNA, while F2/R2 region represented an unrelated region nearby. (**D**) The construction for the E2F1 3′-UTR region containing pmir-GLO luciferase reporters. (**E**) Luciferase activities of pmir-GLO reporters in sh-IGF2BP1/sh-NC-expressing HK2 cells with or without the treatment of LPS for 12 h, as measured by luciferase assay. (**F**) Schematic presentation of the mutation of the m6A region in E2F1 3′-UTR. (**G**) Luciferase activities of pmir-GLO reporters that contained wild-type (WT) or m6A site-mutated (Mut) E2F1 3′-UTR region in HK2 cells following treatment with LPS for 12 h, as measured by luciferase assay. (**H**) Act D chase assays for the WT or mutated-F-Luc-E2F1 fusion mRNA levels in HK2 cells with or without the knockdown of IGF2BP1 following treatment with LPS for 12 h. Statistical analysis was performed using two-way ANOVA (B, C, H) and one-way ANOVA (E, G). **P < 0.01, ***P < 0.001, and ns, non-significance.

We mined the DART-seq data (GSE54365) 56 from an online database RMVar. The m6A peak (chr20: 33675860-33676120) was identified in the 3′-UTR of E2F1 mRNA (chr20: 33675477-33676731), and knocking down METTL3 decreased the reads of this peak (Figure 6A). We also discovered that knocking down METTL3 in HK2 cells decreased m6A enrichment in the F1/R1 region that encompassed the potential m6A site (Figure 6B). Meanwhile, overexpressing METTL3 increased m6A enrichment in the F1/R1 region (Figure 6C). Nonetheless, the m6A-unrelated region (F2/R2) downstream of the F1/R1 region, exhibited no response to the METTL3 expression alteration (Figure 6B & 6C). Similar to previously published findings 49, our data revealed that the m6A methylation in the 3′-UTR region of E2F1 mRNA also occurred in HK2 cells.

Despite the fact that E2F1 mRNA can be m6A methylated, it was unclear whether IGF2BP1 stabilized E2F1 mRNA via the putative m6A region. The E2F1 3′-UTR region containing the m6A site was a cloned downstream of the firefly luciferase coding region of the pmir-GLO plasmids (Figure 6D). Hence, the luciferase activity could directly reflect the function of the m6A site to stabilize mRNA. Induction of IGF2BP1 in HK2 cells was found to significantly boost luciferase activities (Figure 6E). In contrast, silencing IGF2BP1 in HK2 cells showed the opposite effect (Figure 6E). The m6A site in the putative m6A region was further mutated (Figure 6F) to see whether IGF2BP1 enhanced mRNA stability through this region. The mutation of this m6A region decreased basal luciferase activity compared to the WT group (Figure 6G). We confirmed the findings of the luciferase assays (Figure 6D-6G) by quantifying the firefly luciferase-E2F1 3′-UTR fusion mRNA generated from the pmir-GLO plasmids in Act D chase experiments (Figure 6H). Hence, according to our findings, the m6A site at the 3′-UTR region of E2F1 mRNA was vital for IGF2BP1 to stabilize E2F1 mRNA.

### Inhibiting IGF2BP1 is a potential strategy to protect against renal septic injury *in vivo*

**Figure 7:**
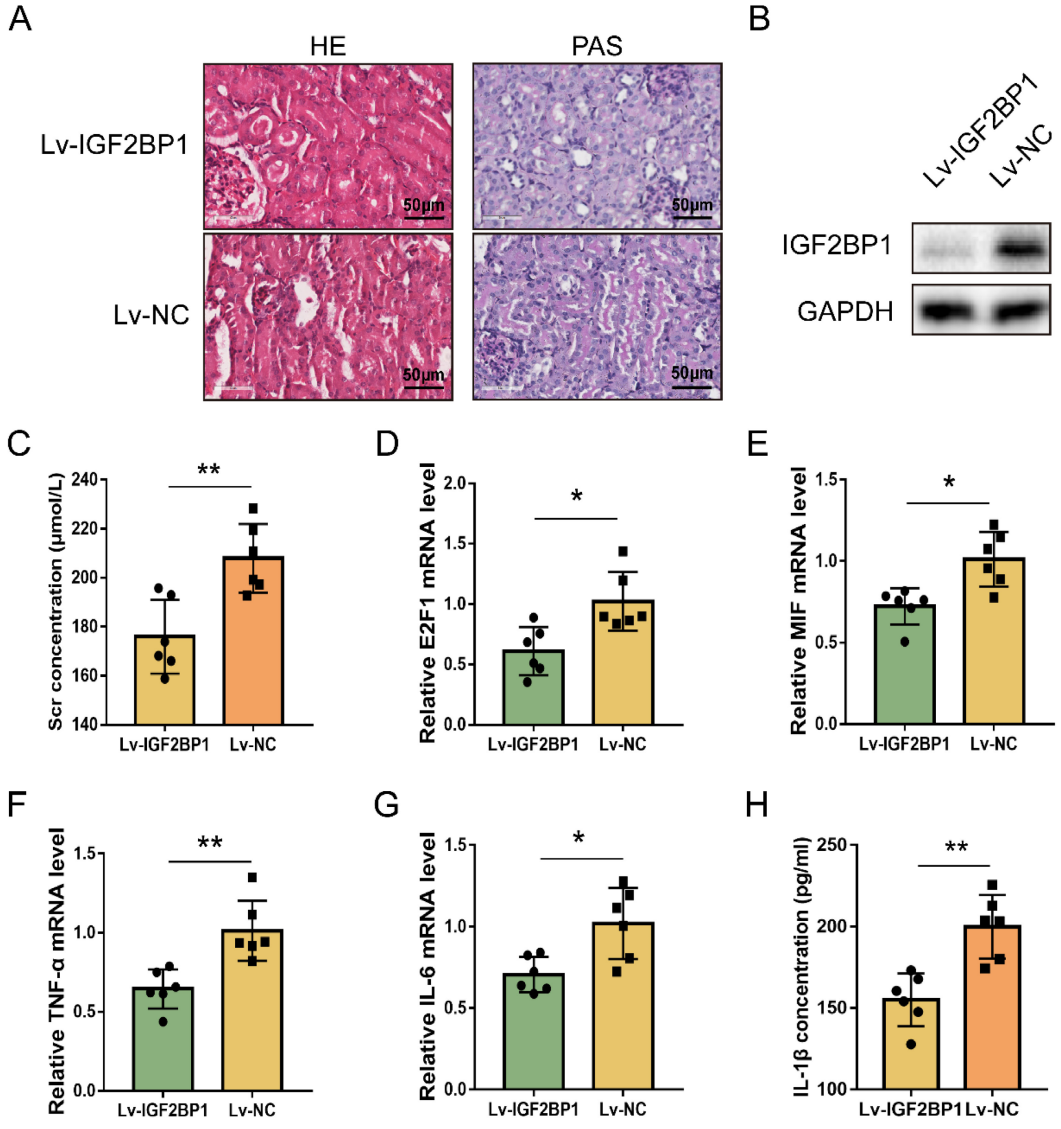
** Inhibiting IGF2BP1 is a potential strategy to protect against renal septic injury *in vivo*.** (**A**) Representative images of H&E and PAS staining of kidney tissues from CLP mice with or without the knockdown of IGF2BP1. Scale bar, 50 μm. (**B**) Expression of IGF2BP1 in the CLP kidney injected with Lv-IGF2BP1 or Lv-NC, as measured by WB. (**C**) Renal function serum creatinine (Scr) level in mice receiving CLP surgery after injection of Lv-IGF2BP1 or Lv-NC. (**D-G**) The expression level of E2F1 mRNA (D), MIF mRNA (E), IL-6 mRNA (F), and TNF-α mRNA (G) in kidney tissues from mice injected with Lv-IGF2BP1 or Lv-NC, as measured by qPCR. (**H**) Serum IL-1β levels in mice injected with Lv-IGF2BP1 or Lv-NC, as measured by ELISA. Group comparisons were performed by t-test (C-H). N = 6/group, *P<0.05, **P < 0.01.

The regulating function of IGF2BP1 in E2F1 and MIF expression during acute renal inflammation *in vivo* was then examined. IGF2BP1 was knocked down through injecting lentiviral vectors into renal parenchyma 57. The glomerular and tubular architecture of mice in the Lv-NC+CLP group was worse in contrast to those in the Lv-IGF2BP1+CLP group (Figure 7A). The knockdown of IGF2BP1 in the renal tissues of mice was verified by WB (Figure 7B). ELISA showed that Scr was improved in mice with IGF2BP1 knockdown (Figure 7C). The level of blood urea nitrogen (BUN) also tended to ameliorate in CLP mice with IGF2BP1 knockdown compared to that in control mice (Figure S6A). Kidney injury molecule-1 (KIM-1) is upregulated at the proximal tubules suffering from kidney injury 58, 59 and serves as a marker for kidney injury 60, 61. As shown in Figure S6B, the mRNA level of KIM-1 was decreased in IGF2BP1 knockdown mice, further suggesting the role of IGF2BP1 in attenuating kidney injury. In addition, E2F1, MIF, IL-6 and TNF-α mRNA levels, were considerably decreased in Lv-IGF2BP1+CLP mice compared to those in control mice (Figure 7D-7G). Similar results were found when measuring the pyroptosis level with IL-1β (Figure 7H). Thus, IGF2BP1 may positively regulate E2F1 and MIF, and inhibiting IGF2BP1 suppressed renal inflammation and pyroptosis during septic AKI.

## Discussion

Clinically, sepsis accounts for around 50% of AKI and is associated with a significant death rate 62. Inflammation, oxidative stress, and cell damage contribute to the development of AKI caused by sepsis. Inflammatory variables play critical roles in PAMPs and DAMPs associated with sepsis-induced AKI. In sepsis-induced AKI, high IL-1β, IL-6, TNF-α, and cell fragments negatively affect renal tubular epithelial cells 63. In AKI, the emphasis of DAMPs is on cell death and the underlying mechanism. Recent research showed that pyroptosis and apoptosis may be the fundamental cell death mechanisms underlying sepsis-induced AKI. Pyroptosis is a well-researched cell death process connected with inflammation. It has been proven that pyroptosis mediated by the NLRP3 inflammasome is also a crucial mechanism of diabetes 64. These results demonstrated that pyroptosis and apoptosis may interact in sepsis-induced AKI. In addition, Caspase4/5/11, a major component of the non-classical route of pyroptosis 65, required additional investigation in sepsis-induced AKI. According to our findings, NLRP3, cleaved Caspase1, and ASC were upregulated in sepsis-induced AKI, which were similar to previous studies.

The RNA m6A level in AKI renal tissues was higher than that in control tissues 66, suggesting a vital role of m6A in AKI, and berberine may alleviate this process 67. Meanwhile, genetic and pharmacological inhibition of METTL3, a typical m6A writer, attenuated renal injury and inflammation 68, 69. IGF2BP1 is one of the common m6A readers but has not been reported in septic AKI; however, studies confirmed that its level could be elevated when induced by LPS and regulate the inflammatory responses 24, 70. In addition, IGF2BP1 was previously reported to promote proliferation *in vitro* and growth *in vivo* in an m6A-dependent manner 71, 72. Apart from the roles in neoplastic diseases, IGF2BP1 is also involved in several nonneoplastic conditions. For example, the role of the METTL3/IGF2BP1/HDAC4 axis was illustrated in sepsis-associated myocardial injury, providing a novel therapeutic strategy 73. In this study, we found high IGF2BP1 expression in septic AKI and that the level of IGF2BP1 was positively correlated with pyroptosis in renal tubular cells. Moreover, IGF2BP1 knockdown inhibited the inflammation level in the kidney. These findings, taken together, indicated that increased IGF2BP1 plays a crucial role in promoting pyroptosis in AKI (Figure S7).

MIF combines with NLRP3 inflammasome and influences its activity, thereby affecting pyroptosis 42, 74, 75. Recently, inhibition of the MIF level has been revealed to reduce inflammation and renal injury in septic AKI mice 44. In the current study, we found that MIF, a subunit of NLRP3 inflammasomes, was indirectly promoted by IGF2BP1 in an m6A-dependent manner. Given the role of IGF2BP1 in promoting the MIF level, we considered that IGF2BP1 was a potent inducer of pyroptosis in renal tubular cells. To the best of our knowledge, neither we nor others have reported that MIF mRNA could be methylated via m6A. However, this did not rule out the possibility that the MIF mRNA was m6A-methylated as the missing of m6A methylation suggested that the quantity of specific changes was insufficient to achieve detection threshold 76. Nonetheless, these negative findings at least indicated that the direct m6A methylation of MIF in HK2 cells was less crucial. Further investigation showed that IGF2BP1 only regulated MIF transcriptionally, suggesting the promotion of MIF protein in AKI might be resulted from prior promotion of its mRNA.

The mRNA of E2F transcription factors (including E2F1, E2F2, and E2F3) can be stabilized by IGF2BP1 49. Interestingly, the E2F1 mRNA encodes the E2F1 protein, a transcription factor capable of stimulating MIF transcription. Thus, the indirect role of IGF2BP1 in promoting MIF-related pyroptosis via E2F1 was established in this study. By analyzing the mRNA of E2F1, we identified a potential m6A site at the 3′-UTR region of E2F1 mRNA. As expected, further exploration revealed that the indirect regulation of MIF by IGF2BP1 was mediated by the m6A methylation of the E2F1 mRNA. E2F1 plays critical immunological roles, as it can not only control the expression of a large number of genes in response to inflammatory stimulation but also contribute to the activation of T cells in response to pathogens 77, 78. In addition, E2F1 has important functions in regulating the cell cycle and apoptosis 79, indicating that IGF2BP1-mediated stability of E2F1 mRNA is effective for promoting pyroptosis and has the potential for accelerating the progression of diseases. Interestingly, deficiency of E2F1 reduces the degree of renal fibrosis and DNA damage 80, and renal fibrosis is implicated in cell dysfunction 81. The bioinformatics analyses, based on the public expression profile determined that E2F1 served importantly in AKI damage repair in two kinds of existing AKI models (ischemia-reperfusion injury and cisplatin) 77. Also, the knockout of E2F1 could significantly protect mice against cisplatin nephrotoxicity under both functional and histological criteria 82. E2F1 had the capacity to promote pyroptosis by elevating MIF expression (Figure S7). Given the strong association between MIF and NLRP3 inflammasomes, the indirect m6A-dependent regulation of MIF via promoting E2F1 plays an important role of IGF2BP1 in acting as a pyroptosis inducer. This assertion also requires further research.

## Conclusion

In this study, the m6A reader, IGF2BP1, was a pyroptosis inducer through indirectly targeting MIF in AKI. The elevation of MIF via E2F1 improved our knowledge that E2F1 and MIF were indispensable for IGF2BP1 to induce pyroptosis. Inhibiting IGF2BP1 could be an alternative strategy in the future to supplement pyroptosis therapy based on small chemical molecules to improve the treatment of AKI.

## Supplementary Material

Supplementary figures and table.

## Figures and Tables

**Table 1:** The clinical characteristics

	Control (n=12)	AKI (n=38)	*P* value
Age	49.42 ± 2.506	52.05 ± 1.659	ns
Male/Female	7/5	21/17	ns
Scr (μmol/L)	70.92 ± 2.047	372.1 ± 22.35	***
BUN (mmol/L)	5.729 ± 0.2615	12.02 ± 0.5017	***

Scr: Serum creatinine; BUN: Blood urea nitrogen. ***P < 0.001, and ns, non-significance.
