# Supplementary material for: Inhibition of IGF2BP1 attenuates renal injury and inflammation by alleviating m6A modifications and E2F1/MIF pathway
Source: Int J Biol Sci. 2023 Jan 1;19(2):593–609. doi: 10.7150/ijbs.78348 (PMC9830505; doi:10.7150/ijbs.78348)
Supplement: Supplementary file 1 — Supplementary figures and table. [file ijbsv19p0593s1.pdf]

Supplementary Figures and Tables

Figure S1. Further investigation of the m6A regulators in CLP mice.

(A) Renal function serum creatinine (Scr) level in CLP and Sham mice. (B) The mRNA levels of m6A regulators between the CLP mice and the Sham ones, as measured by qPCR. Group comparisons were performed by t-test. N = 6/group, **P*<0.05, ****P* < 0.001.

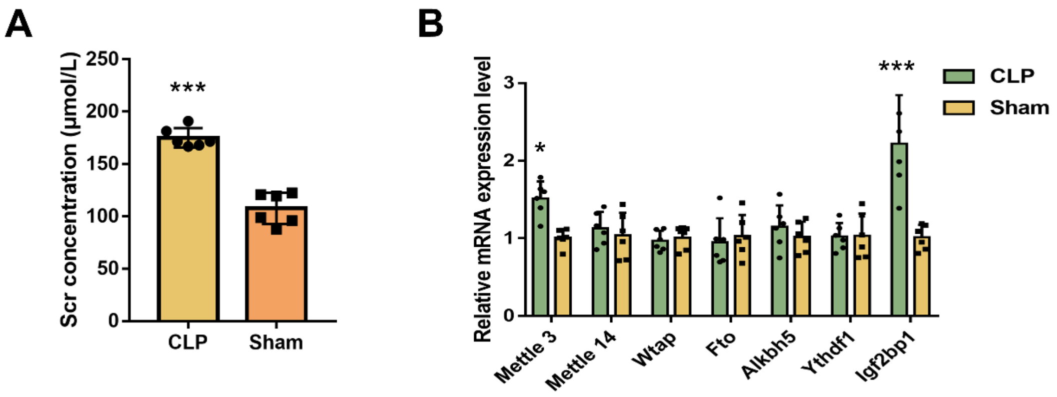

Figure S2. Validation of the inflammatory state of HK2 cells stimulated by LPS. (A) The mRNA levels of IL-6 and TNF-α in HK2 cells following stimulation with different concentrations of LPS for 12 h, as measured by qPCR. (B) The IL-6 and TNF-α levels in the supernatants of HK2 cells following treatment with the indicated concentration of LPS for 12 h, as measured by ELISA. Statistical analysis was performed using two-way ANOVA (A) and t-test (B). *$P < 0.05$, **$P < 0.01$, and ***$P < 0.001$.

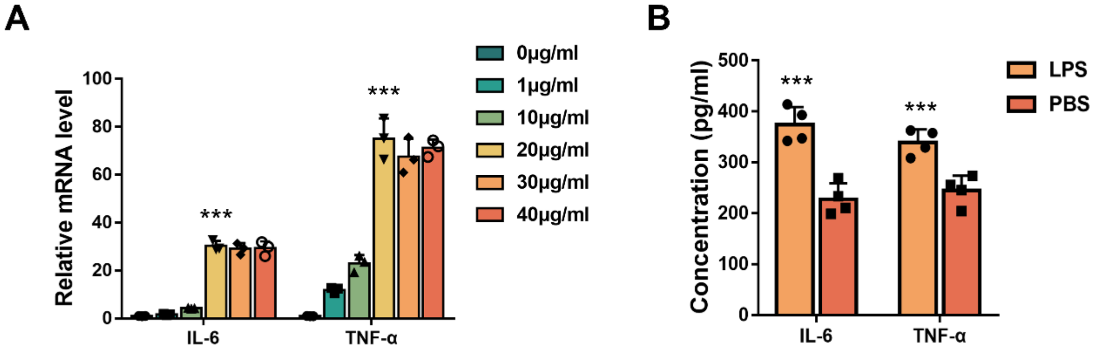

Figure S3. Prediction of the promoter region of MIF using online software.

(A) Prediction of the promoter region of MIF using FPROM. (B) Prediction of the promoter region of MIF using PROMOTER 2.0. (C) Prediction of the promoter region of MIF using TSSG. (D) Prediction of the promoter region of MIF using TSSW.

A FPROM

```
Sequence    1 of    1,  Name: NC_000022.11:23892383-23894582 Homo sapiens chromosome 22, GRCh38.p13 Primary
Length of sequence:      2200
     2 promoter/enhancer(s) are predicted
Promoter Pos:      2011 LDF:   +5.220 TATA box at       1983    +3.372 CACAAAAG
Promoter Pos:      1494 LDF:   +1.403 TATA box at       1463    +6.742 TTTAAAGA
```

B PROMOTER 2.0

Promoter-2.0 Server Output - DTU Health Tech

```
NC_000022.11_23892383-23894582 Homo sapiens chromosome 22, GRCh38.p13 Primary Assembly, 2200 nucleotides

  Position  Score  Likelihood
       400  0.575  Marginal prediction
      1600  1.267  Highly likely prediction
```

C TSSG

```
>NC_000022.11:23892383-23894582 Homo sapiens chromosome 22, GRCh38.p13 Primary
 Length of sequence-      2200
 Threshold for LDF-  4.00
     3 promoter(s)  were predicted
 Pos.:   2015 LDF- 39.82 TATA box predicted at   1982
 Pos.:   1562 LDF-  6.06
 Pos.:    983 LDF-  5.96 TATA box predicted at    953
```

D TSSW

```
>NC_000022.11:23892383-23894582 Homo sapiens chromosome 22, GRCh38.p13 Primary
 Length of sequence-      2200
 Thresholds for TATA+ promoters -  0.45, for TATA-/enhancers -  3.70
     5 promoter/enhancer(s) are predicted
 Enhancer Pos:   1986 LDF- 43.94
 Enhancer Pos:   1605 LDF- 10.78
 Promoter Pos:   1553 LDF- 10.36
 Promoter Pos:   2014 LDF-  3.15 TATA box at   1982    17.13
 Promoter Pos:    984 LDF-  1.34 TATA box at    953    20.73
```

Figure S4. Validation of the conservatism of the binding motif for E2F1 and MIF promoter in other species.

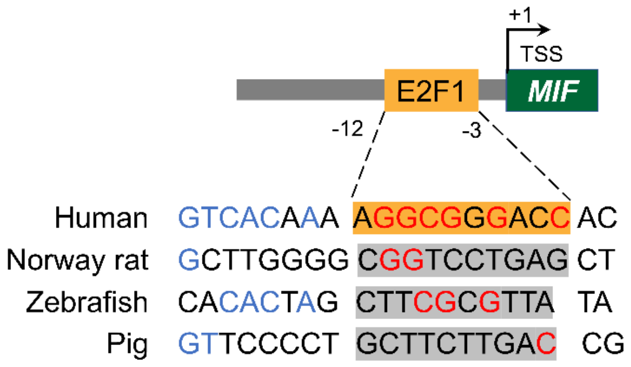

Figure S5. The decline in E2F1 and MIF levels caused by DAA was reversed when IGF2BP1 was overexpressed.

(A&B) The mRNA level of E2F1 (A) and MIF (B) in HK2 cells with IGF2BP1 overexpression and DAA stimulation, as measured by qPCR. Statistical analysis was performed using one-way ANOVA (A&B). **P* < 0.05, ***P* < 0.01, and ****P* < 0.001.

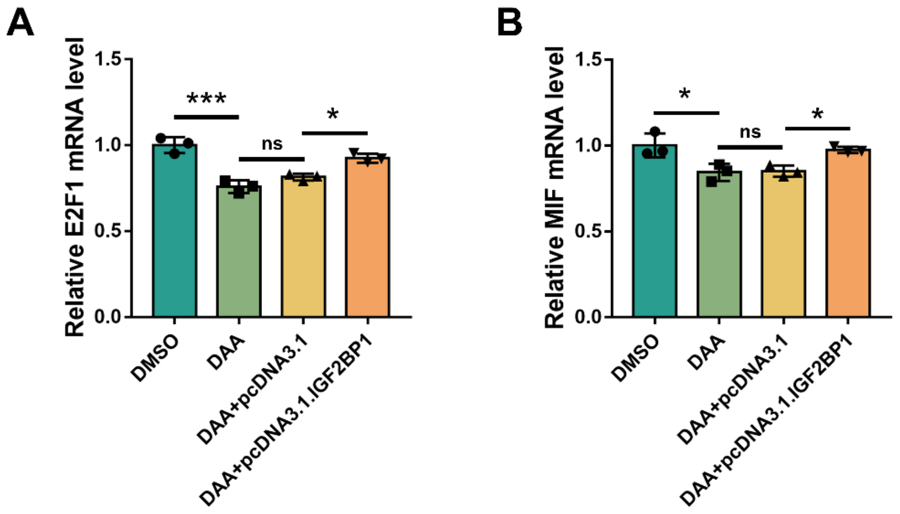

Figure S6. Knockdown of IGF2BP1 ameliorates kidney injury in CLP mice.

(A) The blood urea nitrogen (BUN) level in mice injected with Lv-IGF2BP1 or Lv-NC, as measured by ELISA. (B) The mRNA level of kidney injury marker KIM-1 in kidney tissues from mice injected with Lv-IGF2BP1 or Lv-NC, as measured by qPCR. Group comparisons were performed by t-test (A&B). N = 6/group, **P*<0.05, ***P* < 0.01.

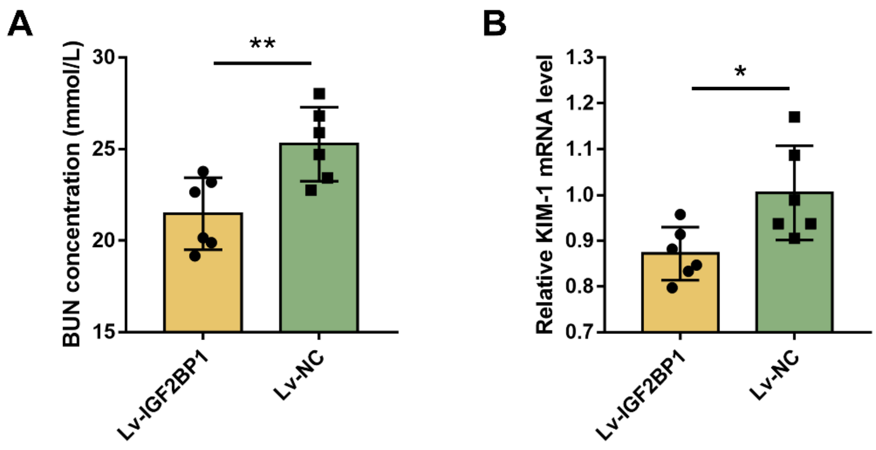

Figure S7. Schematic presentation of the study.

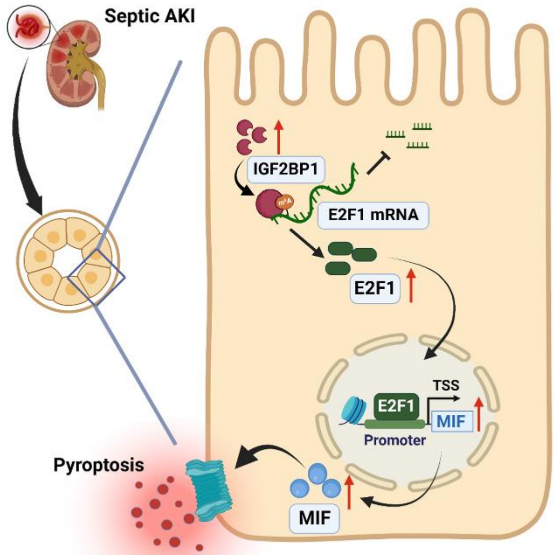

Table S1. The details of primers, probe sequences, and shRNA sequences.

**Primers used for qPCR**		
Gene	5'→3'	
Human		
IGF2BP1	forward	TAGTACCAAGAGACCAGACCC
	reverse	GATTTCTGCCCGTTGTTGTC
E2F1	forward	AGCGGCGCATCTATGACATC
	reverse	GTCAACCCCTCAAGCCGTC
MIF	forward	CGGACAGGGTCTACATCAA
	reverse	CTTAGGCGAAGGTGGAGTT
Mouse		
Igf2bp1	forward	TGAAGGCCATCGAAACTTTCTC
	reverse	ATCGGAGCTGAGGTGGAATA
E2f1	forward	AAGAGCAAACAAGGCCCGAT
	reverse	ACAACAGCGGTTCTTGCTCC
Mif	forward	GTTTCTGTCGGAGCTCAC
	reverse	AGCGAAGGTGGAACCGTTCCA
Tnf-α	forward	CCTGGCCTCTCTACCTTGTTG
	reverse	AGCCTGGTCACCAAATCAGC
Il-6	forward	CTGCAAGAGACTTCCATCCAG
	reverse	AGTGGTATAGACAGGTCTGTTGG
Mettl 3	forward	GAAACAGCTGGACTCGCTTC
	reverse	GGCACGGGACTATCACTACG
Mettl 14	forward	GAGCTGAGAGTGCGGATAGC
	reverse	GCAGATGTATCATAGGAAGCCC
Wtap	forward	GAACCTCTTCCTAAAAAGGTCCG
	reverse	TTAACTCATCCCGTGCCATAAC
Fto	forward	TCACAGCCTCGGTTTAGTTC
	reverse	GCAGGATCAAAGGATTTCAACG
Alkbh5	forward	GCATACGGCCTCAGGACATTA
	reverse	TTCCAATCGCGGTGCATCTAA
Ythdf1	forward	GGACAGTCCAATCCGAGTAACA
	reverse	GTGAGATACGGGATGGGAGG
Kim-1	forward	TCAGAAGAGCAGTCGGTACAAC
	reverse	TGTAGCTGTGGGCCTTGTAGT
**Primers used for ChIP**		
MIF promoter	5'→3'	
	F1	GCGGTGACTTCCCCACTC
	R1	CCTGACTTCTCGGACACCAC
	F2	TGCTAGATGGTCCCCGAGTT
	R2	AAGCCACACCCTAGTCAGGT

**Probe sequences for EMSA**		
Name	5’→3’	
Wt	forward	CAGGCCGATTTCTAGCCGCCAAGTGGAGAACAGGTTGGAGC
	reverse	GCTCCAACCTGTTCTCCACTTGGCGGCTAGAAATCGGCCTG
Mut	forward	CAGGCCGATTTCTAGCATGCAAGTGGAGAACAGGTTGGAGC
	reverse	GCTCCAACCTGTTCTCCACTTGCATGCTAGAAATCGGCCTG

**Sequences of shRNAs**	
Sequences	5'→3'
shMIF	GGGAGAAATAAACGGTTTATT
shIGF2BP1	GCTCCCTATAGCTCCTTTATT
shE2F1	CACTGAATCTGACCACCAATT
shMETTL3	GCTGCACTTCAGACGAATT
shNC	TTCTCCGAACGTGTCACGTTT
